# Are Horizontal Fusional Vergences Comparable When Measured Using a Prism Bar and Synoptophore?

**DOI:** 10.22599/bioj.326

**Published:** 2024-03-22

**Authors:** Shania Haque, Sonia Toor, David Buckley

**Affiliations:** 1 The Eye Centre, Chesterfield Royal Hospital, Chesterfield, UK; 2 Division of Ophthalmology and Orthoptics, Health Sciences School, University of Sheffield, Sheffield, UK

**Keywords:** Fusional vergences, prism bar, synoptophore

## Abstract

**Aim:**

To determine whether horizontal fusional vergences are comparable when measured using a prism bar and synoptophore.

**Methods:**

Thirty two participants (18–23 years) had their blur, break, and recovery points measured for convergence and divergence amplitudes using a prism bar (6 m) and synoptophore. All participants had VA of 0.1 LogMAR or better in either eye, were heterophoric or orthophoric and had binocular single vision. The prism bar target was a 0.2 LogMAR letter. The synoptophore target was the foveal ‘rabbit’ fusion slides. The prism bar was placed over the dominant eye and the testing speed was two seconds per two prism dioptres (Δ), increasing to five seconds per 5Δ when the increments began to increase in 5Δ. Synoptophore testing speed was two seconds per degree.

**Results:**

The synoptophore measured significantly higher convergence break points than the prism bar (Z = 3.37, p = 0.001). No significant differences were found between both tests for divergence break points (Z = 0.99, p = 0.32). However, both tests displayed wide limits of agreement (LoA) when measuring convergence (–24Δ to + 49.59Δ) and divergence break points (–7.70Δ to + 10.19Δ). Differences when measuring convergence and divergence blur and recovery points were not statistically significant.

**Conclusion:**

There was a statistically and clinically significant difference when measuring convergence break points using the prism bar and synoptophore but no significant difference when measuring divergence break points. However, both tests displayed wide LoA when measuring convergence and divergence break points, indicating they should not be used interchangeably in clinic to measure horizontal fusional vergences.

## Introduction

Testing motor fusion is an essential part of a binocular single vision (BSV) assessment, indicating how well a latent deviation is compensated. The quality of motor fusion is represented by fusional vergences, consisting of horizontal, vertical and cyclovergences. Horizontal fusional vergences are most commonly assessed in clinics and consist of convergence and divergence amplitudes.

Horizontal fusional vergences can be measured using several methods. The step vergence method, usually tested using a prism bar, involves phasic fusion and is a fast system driven by retinal disparity (O’Connor & Stephenson 2008). As this is tested in free space, it allows normal seeing conditions with peripheral cues, representing a more natural setting (Wesson 1982). The smooth vergence method, usually tested on the synoptophore, involves tonic fusion and is a slow system driven by prism adaptation (O’Connor & Stephenson 2008). Synoptophore measurements are not taken in free space but have the advantage of being able to assess a patient’s potential for BSV.

Smooth vergence can also be measured using rotary prisms, which consist of two prisms stacked on top of one another with a prismatic effect of zero if stacked base-to-apex. Rotation of the prisms causes the bases to move the opposite way in equal amounts, gradually increasing the prismatic effect. Although rarely used in orthoptic clinics, most of the research comparing the methods to measure smooth fusional vergences involves rotary prisms.

Goss and Becker (2011) compared step and smooth vergence using prism bars and rotary prisms (1/3 m). The prism bar measured a significantly higher convergence blur point (26.7 ± 11.0Δ vs 23.0 ± 10.1Δ), convergence break point (28.9 ± 11.0Δ vs 25.9 ± 9.7Δ), convergence recovery point (16.0 ± 7.5Δ vs 12.6 ± 9.4Δ) and divergence recovery point (12.0 ± 4.1Δ vs 10.9 ± 4.9Δ). They found high coefficients of agreement between both tests, suggesting a weak agreement, and concluded that the two tests cannot be used interchangeably. A similar study by Ciuffreda et al. (2006), also found significantly higher convergence break points with the prism bar (39.1Δ) than rotary prisms (32.3Δ). Differences in divergence break points for both studies were non-significant. They suggested that the higher prism bar measurements could be due to increased input from peripheral vision or because prism bars use a non-continuous scale over-estimating fusional vergences by up to 2Δ or 5Δ. In contrast to these studies, Antona et al. (2008) found rotary prisms produced higher convergence break points (1/3 m: 29.24 ± 8.36Δ vs 28.91 ± 9.09Δ; 6 m: 24.68 ± 7.35Δ vs 23.25 ± 7.68Δ) and divergence break points (1/3 m:15.98 ± 4.29Δ vs 12.14 ± 3.35Δ; 6 m: 9.99 ± 2.36Δ vs 8.63 ± 1.94Δ). However, it was not clear if these differences were statistically significant. Antona et al. (2008) suggested it is easier to achieve higher fusional vergences when prism strength is gradually increased binocularly with rotary prisms. The prism bar test uses asymmetrical vergence and step vergence-type changes in prism demand; this may be more difficult to overcome because only one eye looks through the prism.

As both the prism bar and synoptophore are used to measure fusional vergences in an orthoptic clinic, understanding their comparability is important. However, there is limited research comparing both tests. Fu et al. (2015) compared measurements from the two tests, in 8–15 year olds with intermittent exotropia (IXT) and no strabismus. When measuring convergence, the prism bar and synoptophore produced similar results in both groups (IXT: prism bar 18.65 ± 1.5Δ and synoptophore 22.62 ± 2.15Δ; non-strabismic: prism bar 26.46 ± 1.53Δ and synoptophore 30.19 ± 1.95Δ). However, when measuring divergence, both tests produced similar results in non-strabismic participants (prism bar 8.81 ± 0.32Δ and synoptophore 8.15 ± 0.44Δ), but the prism bar measured a higher fusional vergence than the synoptophore in IXT (prism bar 18.75 ± 0.99Δ and synoptophore 8.98 ± 1.82Δ). As the main purpose of the study was to compare IXT with non-strabismic patients, statistical significance between the prism bar and synoptophore data was not tested. However, the difference in the binocular state during testing may cause a difference in results, as participants were in a state of spontaneous fusion with the prism bar, whereas their deviation was neutralised on the synoptophore. The presence of a strabismus makes it difficult to decipher the true difference in measurements using both tests.

O’Connor and Stephenson (2008) investigated the difference between horizontal fusional vergences measured with a prism bar, rotary prisms and the synoptophore in a group of typical young adults. They found no significant differences between the median values for the distance prism bar (34Δ; interquartile range (IQR): 22, 39), distance rotary prisms (29Δ; IQR: 32, 54) and synoptophore (37Δ; IQR: 24, 54) fusional vergence (sum of convergent and divergent break points) but there was a significant variation on an individual basis, concluding that the tests cannot be used interchangeably. However, the study encountered a problem with ceiling effects when testing with the prism bar. There was no control of testing speeds and a longer viewing time may cause higher values (Ludden & Codina 2013). There was no assessment of blur and recovery points. Lastly, the study did not specify which eye the prism bar was placed over and placing the prism over the non-dominant eye produces larger convergence amplitudes (Hainey 1999; Wesson 1982). Due to these limitations, it is not clear if these tests are interchangeable. The current study aims to address these concerns to determine whether horizontal fusional vergences are comparable when measured using a prism bar and synoptophore.

## Methods

The study adhered to the Declaration of Helsinki. Ethical approval was obtained from the University of Sheffield Research Ethics Committee and written informed consent was obtained from all participants.

Healthy students, aged between 18–25 years were recruited from the University of Sheffield. Criteria for inclusion were (1) best-corrected distance VA of 0.1 LogMAR or better in either eye (2) no manifest/decompensating deviation confirmed using the cover test and Bagolini Glasses (1/3 m and 6 m) and (3) stereopsis of 85” of arc at 40 cm measured using the Frisby stereo test.

This study used a repeated measures design. All participants’ horizontal fusional vergences were measured using a prism bar and synoptophore. The study aimed to record break points and as secondary aims, blur and recovery points.

Testing speed was kept constant using a computerised metronome. The prism bar testing speed was two seconds per 2Δ and increased to five seconds per 5Δ when the increments began to increase in 5Δ. The synoptophore scale is in degrees, so the equivalent testing speed was two seconds per degree (approximately 2Δ in one degree). Testing was always done in the same room with lights on and blinds down to keep lighting consistent. All measurements were obtained by the same examiner (SH), so the instructions given and the method of testing were the same for all participants.

The prism bar was held over the dominant eye. This was found using a quick ocular dominance test. The participant framed a distant object with the thumb and index finger of both hands (like a triangle), with both eyes open. They then closed each eye alternately; the dominant eye was the eye which kept the object contained within the triangle (Shneor & Hochstein 2006).

Confounding variables were limited by counterbalancing the test (prism bar/synoptophore) to be used first and the amplitude (convergence/divergence) to be measured first to limit practice and fatigue effects. Instructions were standardised with no encouragement given, as encouraging a participant on one test more than the other may produce higher results (Fray 2017; Horwood & Toor 2014).

In clinic, the prism strength before the break is recorded with the prism bar, and the exact break point is recorded on the synoptophore. If the present study recorded break points as done in clinic, it may produce lower prism bar values. Therefore, for both tests, the break point was recorded as the point at which diplopia was reported. To prevent ceiling effects, when testing with the prism bar, if the break was not reported at 45Δ an additional prism bar was introduced over the non-dominant eye. For the synoptophore, rather than setting the vergence scale to mid-way to allow for convergence and divergence testing, the scale was always reset to zero to maximise the possible range and prevent ceiling effects. If blur was not reported with either test, then the blur point used for analysis was recorded as equal to the break point. Recovery points were recorded as the point at which single vision was regained.

### Prism bar measurement

The participant’s fusional vergence was measured using a horizontal Clement Clarke prism bar, which increased in intervals of 2Δ to 20Δ, then intervals of 5Δ to 45Δ. The measurement was only taken in the distance (6 m), so the results were comparable to the synoptophore, which simulates distance viewing. All participants viewed a single 0.2 LogMAR letter ‘H’, (on an ETDRS chart, using the Thompson software). The prism strength was increased, until the participant reported blur, then break, and then decreased until they reported recovery. The break point was confirmed by the examiner when no subsequent recovery of motor fusion was seen. The instructions to participants were as follows: to report blur when the letter blurred, report break when the letter became double, and report recovery when the double image re-joined to form a single image.

### Synoptophore measurement

Initially, the foveal simultaneous perception slides (images of a lion and cage) were used to correct the inter-pupillary distance (IPD) and any subjectively measured heterophoria by asking the participant to use the tube to move the lion into the cage. Following this, the foveal motor fusion slides (images of rabbits) were inserted to measure horizontal fusional vergences. Measurements were taken by turning the tubes to converge/diverge until participants reported blur, then break, and then turning them in the opposite direction until they reported recovery, using the same instructions as those for the prism bar method. Additional instructions to the participant were as follows: they were asked if both controls were seen (the rabbit should have a tail and be holding flowers). If one control was not seen, the participant was suppressing, therefore they would not have been used in the experiment. If either of the controls disappeared during the assessment, participants were asked to report this, as this indicated suppression. In this case, their result would be discarded.

### Statistical analysis

All synoptophore values were converted from degrees to Δ, so both tests had the same unit of measurement. For angles below 45° (100Δ), one degree equals approximately 2Δ. For angles over 45° (100Δ) this approximation no longer applies, because when approaching 90°, the number of prism dioptres per degree increases to infinity (Irsch 2015). Therefore, the conversion was calculated using the following formula: 100 × tan (angle in degrees).

Statistical analysis was performed using the Wilcoxon signed-rank test on SPSS. This compared mean convergence and divergence blur, break and recovery points for both tests. Correlations of horizontal fusional vergences detected using both tests were calculated using Spearman’s rank correlation coefficient on SPSS. P-values ≤ 0.05 were considered statistically significant. The agreement between both tests was checked using Bland-Altman on GraphPad Prism-8.

## Results

Thirty two participants were recruited, six male (18.75%) and 26 female (81.25%), with a mean ± standard error (SE) age of 20.22 ± 0.24 years (range: 18–23 years). Nineteen (59.38%) participants were exophoric, eight (25%) were esophoric and five (15.63%) were orthophoric. Twelve (37.5%) participants were left-eye dominant and 20 (62.5%) were right-eye dominant.

Table 1 includes the blur, break and recovery points measured by the prism bar and synoptophore. The Shapiro-Wilk test (GraphPad Prism-8) confirmed the convergence (W = 0.93, p = 0.03) and divergence (W = 0.82, p = 0.0001) break point data was not normally distributed using both tests and therefore a non-parametric test (Wilcoxon signed-rank) was used to analyse data.

**Table 1:** Blur, break and recovery points measured by the prism bar and synoptophore. PB: prism bar; Syn: synoptophore.


TEST	MEAN (Δ)	MEDIAN (Δ)	STANDARD ERROR (Δ)	RANGE (Δ)

PB convergence BLUR	11.78	10	0.89	4.00–25.00

Syn convergence BLUR	13.73	10.51	1.52	5.24–38.39

PB convergence BREAK	18.09	16.00	1.27	6.00–35.00

Syn convergence BREAK	30.89	24.93	3.79	6.99–83.91

PB convergence RECOVERY	14.94	14.00	1.11	4.00–30.00

Syn convergence RECOVERY	20.15	15.84	3.18	1.75–80.98

PB divergence BLUR	6.63	6.00	0.40	2.00–12.00

Syn divergence BLUR	8.05	6.99	0.77	1.75–23.09

PB divergence BREAK	7.19	7.00	0.37	4.00–12.00

Syn divergence BREAK	8.44	7.87	0.71	3.49–23.09

PB divergence RECOVERY	4.69	4.00	0.38	2.00–10.00

Syn divergence RECOVERY	5.47	4.37	0.64	1.75–17.63


### Convergence break point

**Figure 1:**
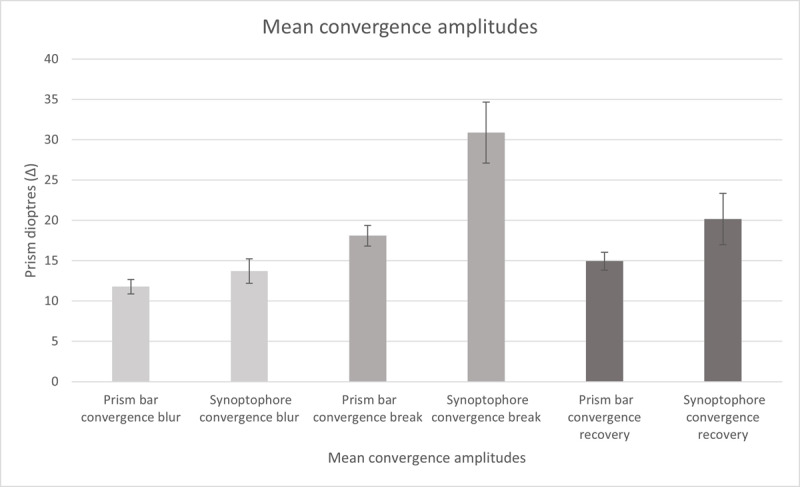
Mean convergence amplitudes for prism bar and synoptophore blur, break and recovery points (Δ). Error bars denote standard error.

Figure 1 displays the convergence data. The mean convergence break point was significantly higher when tested using the synoptophore (30.89 ± 3.79∆) than the prism bar (18.09 ± 1.27∆; Z = 3.37, p = 0.001. Twenty-five (78%) participants had a higher convergence break point on the synoptophore. The difference between the prism bar and synoptophore measurements ranged from –15.56∆ to 62.13∆.

**Figure 2:**
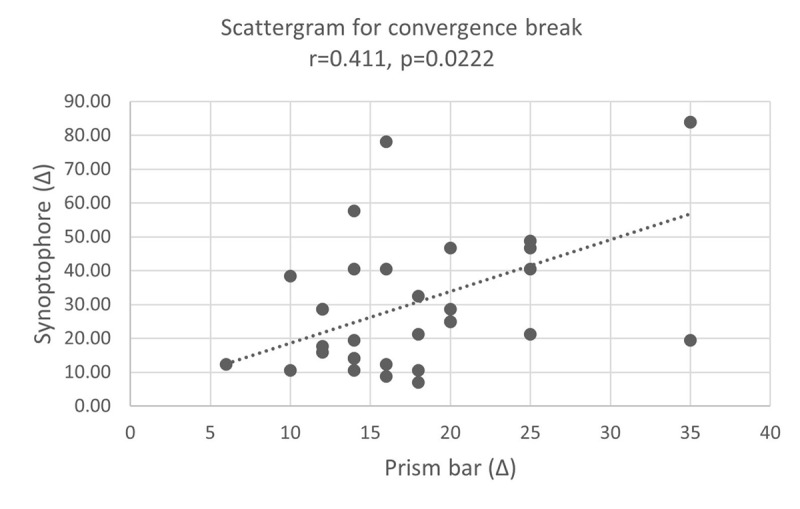
Correlation between prism bar (x-axis) and synoptophore (y-axis) for convergence break points.

A Spearman’s rank correlation coefficient found a significant moderate positive correlation when comparing convergence break points using the prism bar and synoptophore (r = 0.41, p = 0.011, Figure 2).

**Figure 3:**
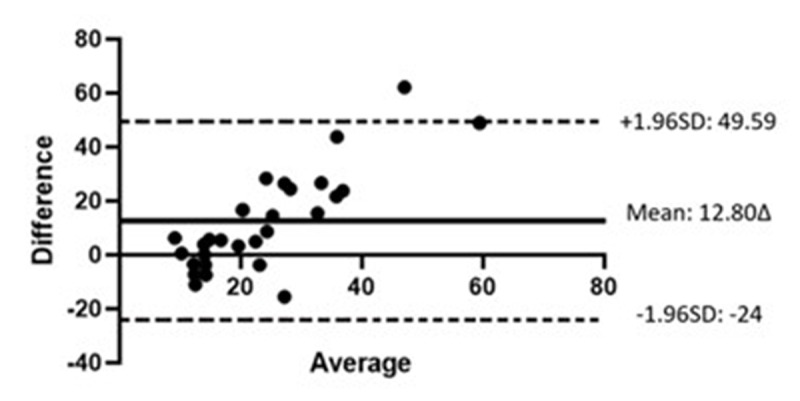
Bland-Altman plot comparing convergence break points when measured with a prism bar and synoptophore. X-axis shows the mean of the two measurements. Y-axis shows the difference between the two values. The solid line represents the mean difference of the measurements from both tests The dashed lines indicate the lower and the upper 95% LoA.

Bland-Altman analysis was performed to analyse the level of agreement between the convergence break points found with the synoptophore and prism bar (Figure 3). The mean difference ± standard deviation (SD) of the difference (12.80Δ ± 18.77Δ) was statistically significantly different from 0 (p < 0.001). The wide 95% limits of agreement (LoA; –24.00Δ to +49.59Δ) is not within the range of clinically acceptable differences and therefore the two tests are not in agreement.

### Convergence blur and recovery points

Twenty six (81%) participants reported blur when tested convergence using the prism bar and 24 (75%) reported blur when tested using the synoptophore. The mean convergence blur point was higher on the synoptophore (13.73 ± 1.52∆) than the prism bar (11.78 ± 0.89∆) but this difference was not statistically significant (Z = 1.40, p = 0.16).

The mean convergence recovery point was also higher on the synoptophore (20.15 ± 3.18Δ) than the prism bar (14.94 ± 1.11Δ) but this difference was also not statistically significant (Z = 1.53, p = 0.13).

### Divergence break points

**Figure 4:**
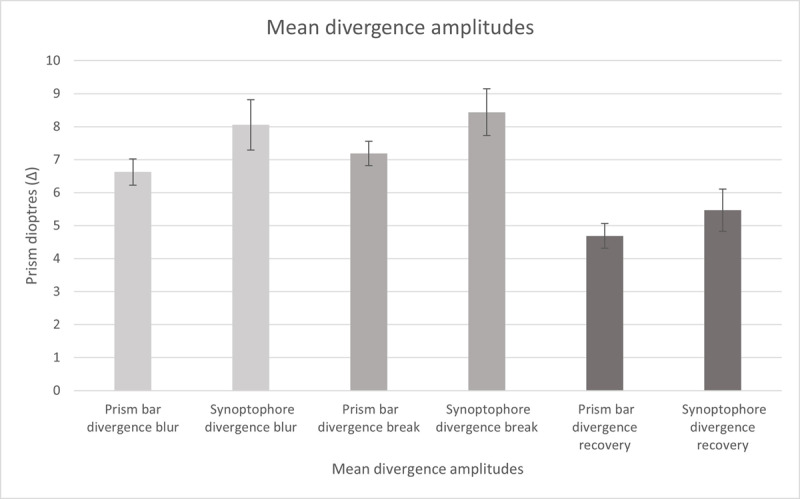
Mean divergence amplitudes for prism bar and synoptophore blur, break and recovery points (Δ). Error bars denote standard error.

Figure 4 displays the divergence data. The mean divergence break point was higher when assessed using the synoptophore (8.44 ± 0.71Δ) than the prism bar (7.19 ± 0.37∆) but this difference was not statistically significant (Z = –0.99, p = 0.32). Unlike the convergence break point, there was a variation in which test produced the higher result, with 17 (53%) scoring higher with the synoptophore and 15 (47%) scoring higher with the prism bar. There was only an average increase of 1.25∆ from the prism bar to synoptophore values, with a difference between measurements ranging from –4.51∆ to 19.09∆.

**Figure 5:**
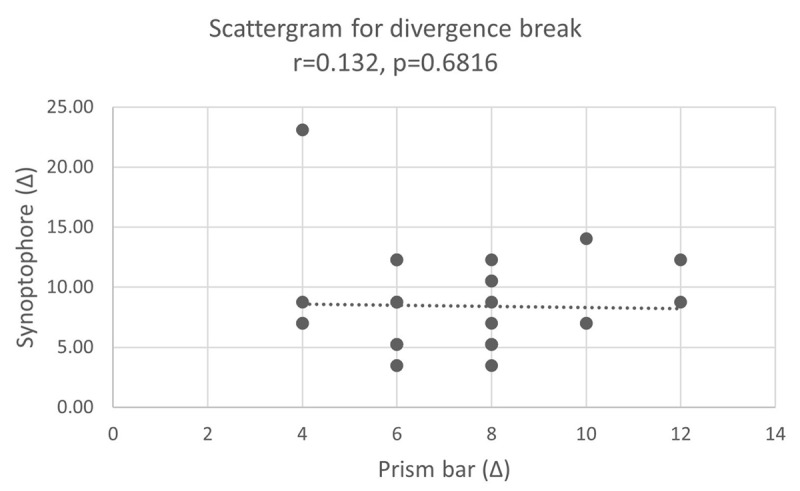
Correlation between prism bar (x-axis) and synoptophore (y-axis) for divergence break points.

A Spearman’s rank correlation coefficient found a non-significant, very weak positive correlation when comparing the mean divergence break points using the prism bar and synoptophore (r = 0.07, p = 0.34, Figure 5).

**Figure 6:**
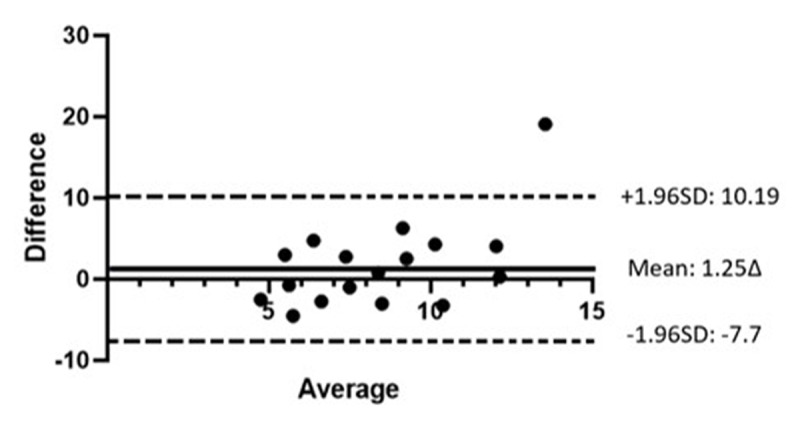
Bland-Altman plot comparing divergence break points when measured with a prism bar and synoptophore. X-axis shows the mean of the two measurements. Y-axis shows the difference between the two values. The solid line represents the mean difference of the measurements from both tests The dashed lines indicate the lower and the upper 95% LoA.

Bland Altman analysis was performed on the divergence break points using the two tests (Figure 6). The mean difference ± SD of the difference (1.25∆ ± 4.56Δ) was statistically significantly different from 0 (p < 0.001). The wide LoA –7.70Δ to +10.19Δ is not within the range of clinically acceptable differences and therefore, the two tests are not in agreement.

### Divergence blur and recovery points

Only seven (21.9%) reported blur when testing divergence using the prism bar and only four (12.5%) reported blur when tested using the synoptophore. The mean divergence blur point was higher on the synoptophore (8.05 ± 0.77Δ) than using the prism bar (6.63 ± 0.40Δ), but this difference was not statistically significant (Z = 1.19, p = 0.23).

Lastly, the mean divergence recovery point was also higher on the synoptophore (5.47 ± 0.64Δ) than using the prism bar (4.69 ± 0.38Δ) and this difference was also not statistically significant (Z = 0.66, p = 0.51).

## Discussion

This study compared horizontal fusional vergences when measured using a prism bar and synoptophore. The synoptophore measured significantly higher convergence break points than the prism bar, with a mean difference of 12.80Δ, which was significantly different from zero with wide LoA. The mean difference between both tests when measuring divergence break points (1.25Δ) was not statistically significant but the Bland-Altman found wide LoA. Although there was a trend for the synoptophore to produce higher measurements, differences when measuring convergence and divergence blur and recovery points were not statistically significant.

In practice, there is natural variability in fusional vergence assessment amongst clinicians, resulting in a typical variability of 3–4Δ (Rouse et al. 2002). The difference in convergence break point between the two tests is therefore also clinically significant. The difference in divergence break points falls within the clinically accepted variability but there was wide LoA. These results are clinically important, indicating the tests are not comparable and should not be used interchangeably when measuring fusional vergences in clinic.

Sheedy and Saladin (1983) stated it is not unusual to find a 10Δ difference between fusional vergences measured using two different tests unless control measures are followed. Limiting proximal convergence was difficult in the current study as the synoptophore is a standardised test and this might explain the higher convergence amplitude seen when tested with the synoptophore. This may also explain why the mean convergence break point on the synoptophore (30.89Δ) resembles a normal 1/3m convergence value (35–40ΔBO), whilst the mean convergence break point when tested with the prism bar (18.09Δ) resembles a normal 6m convergence value (15ΔBO) (Ansons and Davis 2013).

Differences in binocular state could be another factor attributing to higher fusional vergences on the synoptophore. With the prism bar, participants overcame the prism with their heterophoria so exophoric participants may have been biased towards the divergent range and the reverse for those with esophoria, causing variable results (Lança & Rowe 2016). On the synoptophore, any deviations were neutralised, meaning participants were not biased to either range, so this could be considered the true threshold (Jampolsky 1970).

Fu et al. (2015) also compared the horizontal fusional vergences with the synoptophore and prism bar but they found no difference in mean convergence (3.73Δ) and divergence (0.66Δ) break points between the two tests in non-strabismic children, suggesting the two tests are comparable. O’Connor and Stephenson (2008) did not separate convergence and divergence amplitudes and instead compared the median value of the fusional vergence to also find no significant difference between the two tests in the distance. The difference in median fusional vergence (3Δ) was much lower than what we had found in the current study. Their results revealed no consistency on which test produced the highest fusional vergence. This is similar to the divergence amplitude in the current study, whereas 78% of our participants had a higher convergence amplitude when tested with the synoptophore. The difference in results could therefore be due to O’Connor and Stephenson (2008) combining the convergence and divergence amplitudes to investigate fusional vergence. Their results, as well as those of Fu et al. (2015), could also differ from our study as they did not take into consideration possible ceiling effects, the impact of testing speed, target size and ocular dominance, all of which could have impacted the results (Hainey 1999; Ludden & Codina 2013; Rowe 2010; Wesson 1982).

Although smooth vergence was tested with rotary prisms, Antona et al. (2008) did find a higher convergence amplitude when compared to the prism bar in the distance. They stated it is easier to overcome the binocular gradual increase on the continuous scale, compared to the monocular asymmetrical vergence with the prism bar. It is not clear whether the difference was statistically significant, but the small difference in mean convergence break point (1.43Δ) and divergence break point (1.36Δ) is not clinically significant. The difference in results from our study could simply be due to the test used to measure smooth vergence, as rotary prisms produce a clinically significant lower fusional vergence than the synoptophore (O’Connor & Stephenson 2008).

Other studies have found a higher fusional vergence with a step vergence method than a smooth vergence method (Ciuffreda et al. 2006; Goss & Becker 2011; O’Connor & Stephenson 2008). They stated this could be due to the influence of peripheral fusion during free space testing, making the prism bar easier to overcome. Alternatively, it could simply be because the step interval overestimates values. Again, this difference in findings could be due to the use of rotary prisms to assess smooth vergence.

The current study aimed to control all confounding variables to establish if any differences were due to the fusional vergence test used. However, it is impossible to limit proximal convergence due to the nature of the test. There was also a slight variation in target size. The foveal fusion slide was larger, subtending an angle of 2.5 degrees (Haag-Streit 2019), whilst the 0.2 LogMAR letter subtends an angle of 0.03 degrees and larger targets have been found to produce higher fusional vergences (Rowe 2010). It was not possible to analyse the relationship between heterophoria and fusional vergences using both tests, due to the small sample size, unequal spread of deviations and because we did not measure the size of the heterophoria. Future research should measure the heterophoria for each participant to establish whether the difference in convergence break points was due to the heterophoria being neutralised on the synoptophore and not with the prism bar. Despite these limitations, the study design was robust, repeatable, and addressed limitations of previous research in this field.

## Conclusion

The synoptophore measured significantly higher convergence break points than the prism bar. The convergence blur and recovery points and the divergence break, blur and recovery points were all higher when measured with the synoptophore than with the prism bar, but they were not significantly different. However, there were wide LoA for convergence and divergence break points that were not within the range of clinically acceptable. Therefore, the prism bar and synoptophore are not comparable and should not be used interchangeably in clinic to measure horizontal fusional vergences.
